# Evaluation of simple rapid HIV assays and development of national rapid HIV test algorithms in Dar es Salaam, Tanzania

**DOI:** 10.1186/1471-2334-9-19

**Published:** 2009-02-18

**Authors:** Eligius F Lyamuya, Said Aboud, Willy K Urassa, Jaffer Sufi, Judica Mbwana, Faustin Ndugulile, Charles Massambu

**Affiliations:** 1 Department of Microbiology and Immunology, Muhimbilli University of Health and Allied Sciences, Dar es Salaam, Tanzania; 2 Department of Hospital Services, Ministry of Health and Social Welfare, Dar es Salaam, Tanzania

## Abstract

**Background:**

Suitable algorithms based on a combination of two or more simple rapid HIV assays have been shown to have a diagnostic accuracy comparable to double enzyme-linked immunosorbent assay (ELISA) or double ELISA with Western Blot strategies. The aims of this study were to evaluate the performance of five simple rapid HIV assays using whole blood samples from HIV-infected patients, pregnant women, voluntary counseling and testing attendees and blood donors, and to formulate an alternative confirmatory strategy based on rapid HIV testing algorithms suitable for use in Tanzania.

**Methods:**

Five rapid HIV assays: Determine™ HIV-1/2 (Inverness Medical), SD Bioline HIV 1/2 3.0 (Standard Diagnostics Inc.), First Response HIV Card 1–2.0 (PMC Medical India Pvt Ltd), HIV1/2 Stat-Pak Dipstick (Chembio Diagnostic System, Inc) and Uni-Gold™ HIV-1/2 (Trinity Biotech) were evaluated between June and September 2006 using 1433 whole blood samples from hospital patients, pregnant women, voluntary counseling and testing attendees and blood donors. All samples that were reactive on all or any of the five rapid assays and 10% of non-reactive samples were tested on a confirmatory Inno-Lia HIV I/II immunoblot assay (Immunogenetics).

**Results:**

Three hundred and ninety samples were confirmed HIV-1 antibody positive, while 1043 were HIV negative. The sensitivity at initial testing of Determine, SD Bioline and Uni-Gold™ was 100% (95% CI; 99.1–100) while First Response and Stat-Pak had sensitivity of 99.5% (95% CI; 98.2–99.9) and 97.7% (95% CI; 95.7–98.9), respectively, which increased to 100% (95% CI; 99.1–100) on repeat testing. The initial specificity of the Uni-Gold™ assay was 100% (95% CI; 99.6–100) while specificities were 99.6% (95% CI; 99–99.9), 99.4% (95% CI; 98.8–99.7), 99.6% (95% CI; 99–99.9) and 99.8% (95% CI; 99.3–99.9) for Determine, SD Bioline, First Response and Stat-Pak assays, respectively. There was no any sample which was concordantly false positive in Uni-Gold™, Determine and SD Bioline assays.

**Conclusion:**

An alternative confirmatory HIV testing strategy based on initial testing on either SD Bioline or Determine assays followed by testing of reactive samples on the Determine or SD Bioline gave 100% sensitivity (95% CI; 99.1–100) and 100% specificity (95% CI; 96–99.1) with Uni-Gold™ as tiebreaker for discordant results.

## Background

Several types of assays for HIV antibody detection have been developed and promoted for HIV screening and diagnosis. Enzyme-linked immunosorbent assay (ELISA) is the most commonly used technique for screening purposes followed by confirmatory testing, most commonly by using Western blot (WB) assay or a second ELISA based on a different test principle and antigen source in an alternative confirmatory strategy. Simple rapid HIV assays which do not require use of instruments have been widely used especially in resource limited settings including African countries. Currently there are many different commercially available ELISAs, as well as simple rapid HIV assays for detection of antibodies to HIV. Evaluation of various anti-HIV-1 assays using panels of American and European sera have shown that most of these assays have a high sensitivity and specificity [1]. However, studies have shown that some of these assays do not have a similar test performance when used for testing of African sera [2]. It is therefore generally recommended to evaluate HIV assays in the context in which they will be used before adopting them for wide scale use [3,4].

Various combinations of ELISAs as alternative confirmatory strategies have been shown to have the same diagnostic accuracy as the use of antibody ELISA followed by WB assay [5]. In recent years, interest has centred on the use of simple rapid HIV assays, especially in voluntary counselling and testing (VCT) facilities. Suitable algorithms based on a combination of two or more simple rapid assays have been shown to have diagnostic accuracy comparable to double ELISA or double ELISA and WB testing strategies [6-9]. Current initiatives and interventions for prevention of mother-to-child transmission (PMTCT) of HIV-1 require the use of simple rapid assays in VCT services and antenatal clinics aiming at giving same day results. The World Health Organization (WHO) performance characterization of rapid HIV tests shows that a number of assays perform very well using international panels of blood samples. Likewise, new assays entering the diagnostics market address both performance characteristics and operational requirements that allow expansion of access to HIV testing in resource-constrained countries.

The current rapid HIV testing algorithm which is in use in Tanzania was developed 6 years ago [10]. This serial testing algorithm is based on the use of Capillus HIV-1/2 assay (Trinity Biotech, Ireland) for screening followed by confirmatory testing of reactive samples by Determine™ HIV-1/2 assay (Abbott Diagnostics, UK) [10]. Capillus assay requires cold storage, making it unsuitable for use in hard to reach areas where electricity is not available or in settings where power outages are frequent. The cold chain dependency for Capillus assay coupled with availability on the market, of newer HIV rapid tests that are cheaper, do not require cold storage and have acceptable performance characteristics compelled the Ministry of Health and Social Welfare to embark on evaluation of some of these rapid HIV assays aiming at developing alternative algorithms for use in Tanzania. Furthermore, there is an urgent need within the country for a suitable algorithm(s) that meet the demand for the scale up of access to HIV screening, diagnosis, treatment and care in line with the Ministry of Health and Social Welfare Strategic Plan for Prevention, Treatment and Care for HIV/AIDS.

The aims of this study were to evaluate the performance of five simple rapid HIV assays using whole blood samples from hospital patients, pregnant women, VCT attendees and blood donors, and to formulate an alternative cost-beneficial confirmatory strategy based on rapid HIV testing algorithms suitable for use in Tanzania.

## Methods

### Selection of Rapid HIV Assays for Evaluation

The rapid HIV assays which were included in the evaluation were selected according to the following WHO recommended criteria: cost per test (< 2 US$), sensitivity (100%), specificity (≥ 99%), additional requirements for items not provided in the kit, experience of use of the kit in the African region, test kit registered in the country, sample type requirements (whole blood, plasma or serum), shelf life (9 months and above), experience of use of the kit in the country and kit storage requirements (2–30°C). Eighteen rapid HIV assays were subjected to the selection criteria. The selection was made by an Evaluation Technical Committee and approved by a Rapid Test Kit (RTK) Evaluation Management Committee appointed by the Ministry of Health and Social Welfare. Five simple rapid HIV assays that included Determine™ HIV-1/2 (Inverness Medical Japan Co. Ltd, Japan), SD Bioline HIV 1/2 3.0 (Standard Diagnostics Inc., Kyonggi-do, Korea), First Response HIV Card 1–2.0 (PMC Medical India Pvt Ltd, Daman, India), HIV1/2 Stat-Pak Dipstick (Chembio Diagnostic System, Inc., New York, USA) and Uni-Gold™ HIV-1/2 (Trinity Biotech, Wicklow, Ireland) were selected for evaluation. These are qualitative assays for the detection of antibodies (IgG, IgM, IgA) specific to HIV-1 and HIV-2 simultaneously in human serum, plasma or whole blood.

### Collection of blood samples

Whole blood samples were collected consecutively within the City of Dar es Salaam from the Pastoral AIDS services of the Archdiocese of Dar es Salaam (PASADA) Care and Treatment Clinic, the African Medical Research Foundation (AMREF) and Muhimbili Health Information Centre (MHIC) VCT clinics and from the Muhimbili National Hospital (MNH) PMTCT clinic and Blood Bank. PASADA and MHIC are health facility/hospital based VCT sites and therefore have been clustered into one category; MNH blood bank represents a setting for HIV screening for blood; MNH PMTCT is a site for screening pregnant women and AMREF is a typical stand-alone VCT. At each site the left over blood specimens from routine testing were collected without patient/client identifiers and were used for the evaluation. At the end of the clinic day, the unlabeled blood specimens were collected from the individual sites by the designated laboratory technologist from the National HIV Reference Laboratory (NHRL) in the Department of Microbiology and Immunology, Muhimbili University of Health and Allied Sciences (MUHAS) who signed a register and dispatch book to ensure accountability of specimens. All specimens collected every day were recorded in the first register book (which included the date and time of specimen collection, serial number, initials of site of collection and pick up personnel) using assigned serial numbers. Following receipt at the NHRL, every specimen was logged in the second register book (which included the date and time of specimen delivery, laboratory ID number, initials of the receiving and testing personnel) and were assigned a laboratory identification number before being given to designated laboratory technologists for testing. A total of 1649 whole blood samples were collected between June and September 2006: 324 from patients attending PASADA clinic, 121 from pregnant women attending MNH PMTCT, 602 and 311 from AMREF and MHIC VCT clinics, respectively, and 291 from blood donors. The HIV serostatus of samples used was unknown prior the evaluation.

### Rapid HIV testing

A trained laboratory technologist performed one type of rapid HIV assay on all blood samples submitted for evaluation each day. Each blood sample was tested using the listed rapid HIV assay in the NHRL, Department of Microbiology and Immunology, MUHAS. Testing on each assay was performed using whole blood according to manufacturer's instructions of individual test kits. HIV results of individual samples were recorded after reading by two different laboratory technologists according to manufacturer's criteria for interpretation of positive, negative or inconclusive results. Repeat testing was done on any sample that gave invalid/equivocal result according to manufacturer's interpretation of results. All results were checked by the testing laboratory technologists and subsequently verified by a clinical microbiologist. Every testing laboratory technologist independently submitted the rapid HIV results on daily basis to a designated senior lab technologist who compared with results from the other four assays under evaluation and would inform the testing technologist to do repeat testing if any result was discordant. Repeat testing results were also recorded. Inno-Lia HIV I/II immunoblot assay (Immunogenetics) was used as a reference method. All samples that were reactive on all or any of the five HIV rapid assays and 10% of non-reactive samples randomly selected were confirmed on the Inno-Lia HIV I/II assay. A total of 1649 samples were tested from different sites and 199 samples tested in the first two days of evaluation were considered as pilot samples. These included 29 samples from PASADA, 81 from AMREF, 35 from MHIC, 23 from MNH PMTCT and 31 from MNH Blood bank. Data on performance appraisal done by testing laboratory technologists based on the following characteristics in the check list: number of steps in the test procedure, clarity of kit instructions, kit and reagent packaging and labeling, and ease of performance, as used in the previous study, [10] were also obtained.

### Criteria for inclusion into the National Algorithm

The criteria for inclusion of an individual rapid HIV assay into the National algorithm were initial sensitivity of 100% and initial specificity of ≥ 99%.

### Ethical consideration

The study was carried out in line with existing ethical guidelines. Ethical clearance was obtained from the Muhimbili University of Health and Allied Sciences Research and Publications Committee. Permission to conduct this evaluation was given by the Ministry of Health and Social Welfare, Tanzania. Samples were only identified by assigned identification numbers and were not linked to client identifiers, and thus ensuring confidentiality.

### Statistical analyses

One hundred and ninety nine pilot samples and those that gave inconclusive and/or western blot indeterminate (17) test results were excluded. Epi Info™ program version 6.7 was used for analysis. Sensitivity and specificity with 95% confidence intervals (CI) of individual rapid HIV assay and combinations of various assays were determined. Positive predictive value (PPV) and negative predictive value (NPV) (with 95% CI) were also determined.

## Results

Of a total of 1433 samples that were tested and included in the final analysis, 390 were confirmed HIV-1 antibody positive by the Inno-Lia and 1043 were HIV negative. The sensitivity and specificity of the five evaluated assays are presented in Table 1. On initial testing, Determine, SD Bioline and Uni-Gold™ each had sensitivity of 100% (95% CI; 99.1–100) while the initial specificity of the Uni-Gold™ assay was 100% (95% CI; 99.6–100). Of the five assays evaluated, HIV 1/2 Stat-Pack Dipstick had comparatively lowest sensitivity and specificity parameters on initial testing. However, after repeat testing performance increased to 100% for both sensitivity and specificity. The performance of First Response was also comparatively low on initial testing, and only sensitivity increased to 100% on repeat testing. There was no any sample which was concordantly false positive in the above three assays.

**Table 1:** Summary of Sensitivity and Specificity of the Rapid HIV Assays Evaluated

Assay	Sensitivity (n = 390)				Specificity (n = 1043)			
	
	Initial testing		Repeat testing		Initial testing		Repeat testing	
	Reactive	% (95% CI)	Reactive	% (95% CI)	Non-reactive	% (95% CI)	Non-reactive	% (95% CI)
Determine	390	100 (99.1–100)	-	-	1039	99.6 (99–99.9)	1041	99.8 (99.3–100)
SD Bioline	390	100 (99.1–100)	-	-	1037	99.4 (98.8–99.7)	1041	99.8 (99.3–99.9)
Uni-Gold™	390	100 (99.1–100)	-	-	1043	100 (99.6–100)	-	-
First Response	388	99.5 (98.2–99.9)	390	100 (99.1–100)	1039	99.6 (99–99.9)	1040	99.7 (99.2–99.9)
Stat-Pak Dipstick	381	97.7 (95.7–98.9)	390	100 (99.1–100)	1041	99.8 (99.3–99.9)	1043	100 (99.6–100)

CI, confidence interval

The sensitivity and specificity of the assays using whole blood samples from various sites are shown in Table 2. Uni-Gold™ had the best performance characteristics with 100% sensitivity and 100% specificity on samples from any of the sites. The Determine, SD Bioline and Uni-Gold™ rapid HIV assays had sensitivity of 100% on samples from each of these sites. All assays had sensitivity of 100% and specificity of 100% on samples from the antenatal clinic at MNH. None of the 10% nonreactive samples randomly selected were positive in the Inno-lia confirmatory antibody assay.

**Table 2:** Performance characteristics of HIV rapid tests according to the samples collected from the sites

Assay	Site							
	
	PASADA + MHIC		MNH Blood bank		MNH PMTCT		AMREF	
	Sensitivity n (%) 95%CI	Specificity n (%) 95%CI	Sensitivity n (%) 95%CI	Specificity n (%) 95%CI	Sensitivity n (%) 95%CI	Specificity n (%) 95%CI	Sensitivity n (%) 95%CI	Specificity n (%) 95%CI
Determine	322/322 (100) 98.9–100	239/241 (99.2) 97.0–99.9	12/12 (100) 73.5–100	246/247 (99.6) 97.8–100	19/19 (100) 82.4–100	82/82 (100) 95.6–100	37/37 (100) 90.5–100	472/473 (99.8) 98.8–100
SD Bioline	322/322 (100) 98.9–100	237/2471 (100) 95.8–99.6	12/12 (100) 73.5–100	245/247 (99.2) 97.1–99.9	19/19 (100) 82.4–100	82/82 (100) 95.6–100	37/37 (100) 90.5–100	473/473 (100) 99.2–100
Uni-Gold™	322/322 (100) 98.9–100	241/241 (100) 98.5–100	12/12 (100) 73.5–100	247/247 (100) 98.5–100	19/19 (100) 82.4–100	82/82 (100) 95.6–100	37/37 (100) 90.5–100	473/473 (100) 99.2–100
First Response	321/322 (99.7) 98.3–100	238/241 (98.8) 96.4–99.7	11/12 (91.7) 61.5–99.8	246/247 (99.6) 97.8–100	19/19 (100) 82.4–100	82/82 (100) 95.6–100	37/37 (100) 90.5–100	473/473 (100) 99.2–100
Stat-Pak Dipstick	316/322 (98.1) 96–99.3	238/241 (98.8) 96.4–99.7	12/12 (100) 73.5–100	247/247 (100) 98.5–100	19/19 (100) 82.4–100	82/82 (100) 95.6–100	34/37 (91.9) 78.1–98.3	472/473 (98.8) 98.8–100

The positive predictive values (PPV) were 99% (95% CI; 97.4–99.7%) for the Determine, 98.5% (95% CI; 96.7–99.4%) for SD Bioline HIV-1/2 3.0, 100% (95% CI; 99.1–100%) for Uni-Gold™, 98.5% (95% CI; 96.7–99.4%) for First Response and 97.2% (95% CI; 95–98.6%) for Stat-Pak Dipstick assays. The negative predictive value (NPV) at initial testing was 100% (95% CI; 99.6–100%) except First Response (99.8%, 95% CI; 99.3–99.9%) and Stat-Pak Dipstick (99.1%, 95% CI; 98.4–99.6%) assays.

Sensitivity, specificity and cost of each of the various combinations of the rapid HIV assays evaluated in an alternative confirmatory testing strategy are shown in Table 3. Several combinations of HIV rapid assays showed sensitivity and specificity of 100%. The cost of an algorithm is based on the cost of screening a sample on the first rapid HIV assay and confirmation of a reactive sample on the second rapid HIV assay.

**Table 3:** Sensitivity (n = 390), specificity (n = 1043) and cost of various combinations of rapid HIV assays

S/N	1^st ^Assay	2^nd ^Assay	Sensitivity % (95% CI)	Specificity % (95% CI)	Cost per algorithm (US$)
1	SD Bioline	First Response	100 (99.1–100)	99.9** (99.5–99.9)	0.47 + 0.65 = 1.12
					
2	First Response***	SD Bioline	99.5 (98.2–99.9)	100 (96–99.1)	0.65 + 0.47 = 1.12
3	SD Bioline	Determine	100 (99.1–100)	100* (96–99.1)	0.47 + 0.80 = 1.27
4	Determine	SD Bioline	100 (99.1–100)	100* (96–99.1)	0.80 + 0.47 = 1.27
5	SD Bioline	Stat-Pak Dipstick	100 (99.1–100)	100* (96–99.1)	0.47 + 0.80 = 1.27
6	Stat-Pak Dipstick***	SD Bioline	97.9 (96–99.1)	100 (96–99.1)	0.80 + 0.47 = 1.27
7	Determine	First Response	100 (99.1–100)	100* (96–99.1)	0.80 + 0.65 = 1.45
8	First Response***	Determine	99.5 (98.2–99.9)	99.9 (99.5–99.9)	0.65 + 0.80 = 1.45
9	First Response***	Stat-Pak Dipstick	99.5 (98.2–99.9)	100 (96–99.1)	0.65 + 0.80 = 1.45
10	Stat-Pak Dipstick***	First Response	97.9 (96–99.1)	100 (96–99.1)	0.80 + 0.65 = 1.45
11	Determine	Stat-Pak Dipstick	100 (99.1–100)	100* (96–99.1)	0.80 + 0.80 = 1.60
12	Stat-Pak Dipstick***	Determine	97.9 (96–99.1)	100 (96–99.1)	0.80 + 0.80 = 1.60
13	SD Bioline	Uni-Gold™	100 (99.1–100)	100* (96–99.1)	0.47 + 1.80 = 2.27
14	Uni-Gold™	SD Bioline	100 (99.1–100)	100* (96–99.1)	1.80 + 0.47 = 2.27
15	Uni-Gold™	First Response	100 (99.1–100)	100* (96–99.1)	1.80 + 0.65 = 2.45
16	First Response***	Uni-Gold™	99.5 (98.2–99.9)	100 (96–99.1)	0.65 + 1.80 = 2.45
17	Uni-Gold™	Stat-Pak Dipstick	100 (99.1–100)	100* (96–99.1)	1.80 + 0.80**** = 2.6
18	Stat-Pak Dipstick***	Uni-Gold™	97.9 (96–99.1)	100 (96–99.1)	0.80 + 1.80 = 2.60
19	Determine	Uni-Gold™	100 (99.1–100)	100* (96–99.1)	0.80 + 1.80 = 2.60
20	Uni-Gold™	Determine	100 (99.1–100)	100* (96–99.1)	1.80 + 0.80 = 2.60

*Combination of the two assays had no concordant false positive results.

**One sample was repeatedly concordantly false positive in the two assays.

***The first test had initial suboptimal sensitivity (< 100%).

****Cost of Stat-Pak Dipstick is indicated as a range 0.8–0.95 US$. The best case scenario of the lowest possible cost for this test was assumed in computing the cost for algorithms involving use of Stat-Pak Dipstick.

Performance appraisal of HIV rapid assays by testing laboratory technologists revealed that SD Bioline, Determine, Uni-Gold™ and First Response assays had similar high score while Stat-Pak had a low score. Ease of performance for SD Bioline assay was reported by testing laboratory technologists to be very easy compared to the other four assays evaluated.

## Discussion

This evaluation was based on the testing of whole blood samples on the five selected rapid HIV assays. Determine, SD Bioline and Uni-Gold™assays had initial sensitivity of 100% (95% CI; 99.1–100) while the initial specificity of the Uni-Gold™ assay was 100% (95% CI; 99.6–100). Overall the performance characteristics of First Response and HIV 1/2 Stat-Pak assays were lower compared to those of Determine, SD Bioline and Uni-Gold™ assays.

The findings of sensitivity of 100% and specificity of 99.4% for SD Bioline in the present evaluation are in agreement with results reported elsewhere [12]. The findings of 100% sensitivity for Determine and Uni-Gold™ assays found in this evaluation were consistent with findings documented in other studies [10,13,14]. The 100% specificity for Uni-Gold™ assay is in contrast with findings from other studies [13,14]. In a previous study that was conducted in Uganda [14], blood donor samples from low HIV prevalence population were used [14] compared to blood samples from different groups including high prevalence population such as hospital patients and pregnant women. In the present evaluation the sensitivity of First Response was lower [(99.5%) 95% CI; 98.2–99.9] than that reported by the manufacturer (100%) while the specificity in the present study [(99.6%) 95% CI 99–99.9%] was higher that that reported by the manufacturer (99.18%). The sensitivity of 97.7% for Stat-Pak assay is lower compared to 100% sensitivity found though the specificity is comparable with that reported from a study done in another setting [14]. Variations in performance of rapid HIV assays in different settings especially with different levels of antigenic challenge are well known. In addition, differences in the performance may relate in part to their inability to detect low antibody titres in people taking antiretroviral therapy with low viral loads. However, in the present study, no specific information was solicited regarding the use of antiretroviral drugs making it difficult to explain the lowest performance of some of the rapid HIV assays evaluated.

Of the five rapid HIV assays evaluated, Uni-Gold™, Determine and SD Bioline were shown to have the required sensitivity and specificity for inclusion into the National rapid HIV testing algorithm. It is possible to have different algorithms based on different combinations of Determine, SD Bioline and Uni-Gold™ assays. From the site specific performance characteristics of the tests, it would appear that a combination comprising of Uni-Gold™ as a first test and Determine or SD Bioline as a second test would be suitable in all settings. On the other hand, a combination comprising of SD Bioline followed by Uni-Gold™ would possibly be useful, in a descending order of priority in: PMTCT and stand alone VCT (equally); facility-based VCT; and blood bank. A combination comprising of Determine followed by Uni-Gold™ would possibly be useful, in a descending order of priority in: PMTCT, stand alone VCT, blood bank and facility based VCT while a combination involving use of SD Bioline followed by Determine would be useful in facility-based VCT, PMTCT setting and stand alone VCT settings. Since all assays have sensitivity of 100%, in the event of shortage of any of the three assays, any available assay (among the three) can be used as the first assay. Determine is one of the assays in the currently used algorithm while SD Bioline is licensed for use in the country. In view of the present results showing acceptable performance characteristics of these three assays and taking into account the multiple interventions for treatment, control and prevention by different partners, it is prudent to consider introducing more than one algorithm for use in the country. In the event that one of the tests is withdrawn from the market, the other algorithm(s) can continue to be used without disrupting service provision in various HIV testing programs.

It is apparent that strategies involving the use of Uni-Gold™ as first or second assay are much more expensive compared to those which use combinations of the other assays. The prices for these tests were obtained from the WHO bulk procurement scheme [15]. Taking into account the criteria for inclusion into the National algorithm and the element of cost, the possible rapid HIV serial testing algorithms are: screening by SD Bioline followed by confirmatory testing of reactive samples by Determine with Uni-Gold™ assay as a tiebreaker for discordant results. The sensitivity and specificity of this strategy is 100%. A strategy based on screening by Determine followed by confirmatory testing of reactive samples by SD Bioline with Uni-Gold™ assay as tiebreaker for discordant results also has a sensitivity of 100% and a specificity of 100%. It would be cheaper to use the former (involving initial screening with SD Bioline) than the later algorithm when serial HIV testing is performed because SD Bioline is relatively cheaper than Determine assay. However, if testing is done in parallel, the cost would be similar for both algorithms. An algorithm based on screening by SD Bioline and confirmatory testing of reactive samples by Stat-Pak Dipstick assay would have a similar cost to that of the above named two algorithms but the initial sensitivity of Stat-Pak Dipstick was 97.7%, which, according to the protocol did not qualify it for inclusion into the National algorithm. The cheapest assay combinations are those involving the use of SD Bioline and First Response assays, with SD Bioline as the first assay followed by First Response as the second assay or vice versa. This algorithm, however, is associated with a concordant false positive reactivity on the two assays as well as the fact that First Response had initial sensitivity of 99.5%. It should be noted that the cost of rapid HIV testing algorithm can change to due to changes in prices of the test kits. A rapid HIV testing strategy that is consistent with recommendations by WHO [16] is essential for promoting HIV care, treatment and prevention programs.

Results from the current evaluation indicate that a number of assays have good performance characteristics suggesting that considering additional operational characteristics other than sensitivity and specificity would be useful in developing a national rapid HIV testing algorithm for Tanzania. This aspect was taken into account in the current evaluation during selection of the tests for inclusion into the evaluation as well as assessment of additional characteristics.

## Conclusion

An alternative confirmatory HIV testing strategy based on initial testing on either SD Bioline or Determine assays followed by testing of reactive samples on the Determine or SD Bioline gave 100% sensitivity (95% CI; 99.1–100) and 100% specificity (95% CI; 96–99.1) with Uni-Gold™ assay as tie breaker for discordant results.

## Competing interests

The conclusions and opinions expressed in this paper are those of the authors and do not necessarily reflect those of the funding agencies and participating institutions. The authors declare that they have no competing interests.

## Authors' contributions

EFL participated in study design, coordination and manuscript writing. SA participated in study design, coordination, statistical analysis and manuscript writing. WKU participated in study design, coordination and statistical analysis. JS participated in the study design, coordination and supervision of laboratory testing. JM participated in the study design, coordination and supervision of laboratory testing. FN participated in the conception, study design and coordination. CM participated in the conception, study design and coordination.

## Pre-publication history

The pre-publication history for this paper can be accessed here:

http://www.biomedcentral.com/1471-2334/9/19/prepub
